# Free-Standing rGO-CNT Nanocomposites with Excellent Rate Capability and Cycling Stability for Na_2_SO_4_ Aqueous Electrolyte Supercapacitors

**DOI:** 10.3390/nano11061420

**Published:** 2021-05-28

**Authors:** Xiaohan Du, Zhen Qin, Zijiong Li

**Affiliations:** 1 School of Physics & Electronic Engineering, North China University of Water Resources & Electric Power, Zhengzhou 450045, China; duxiaohan@ncwu.edu.cn (X.D.); qinzhen@ncwu.edu.cn (Z.Q.); 2 School of Physics & Electronic Engineering, Zhengzhou University of Light Industry, Zhengzhou 450002, China

**Keywords:** rGO-CNT nanocomposite, synergistic enhancement effect, excellent electrochemical properties, supercapacitors

## Abstract

Facing the increasing demand for various renewable energy storage devices and wearable and portable energy storage systems, the research on electrode materials with low costs and high energy densities has attracted great attention. Herein, free-standing rGO-CNT nanocomposites have been successfully synthesized by a facile hydrothermal method, in which the hierarchical porous network nanostructure is synergistically assembled by rGO nanosheets and CNT with interlaced network distribution. The rGO-CNT composite electrodes with synergistic enhancement of rGO and CNT exhibit high specific capacitance, excellent rate capability, exceptional conductivity and outstanding long-term cycling stability, especially for the optimal rGO-CNT_30_ electrode. Applied to a symmetric supercapacitor systems (SSS) assembled with an rGO-CNT_30_ electrode and with 1 M Na_2_SO_4_ aqueous solution as the electrolyte, the SSS possesses a high energy density of 12.29 W h kg^−1^ and an outstanding cycling stability, with 91.42% of initial specific capacitance after 18,000 cycles. Results from these electrochemical properties suggest that the rGO-CNT_30_ nanocomposite electrode is a promising candidate for the development of flexible and lightweight high-performance supercapacitors.

## 1. Introduction

With the continuous growth of population and the rapid development of economic globalization, the energy crisis and environmental pollution caused by the rapid consumption of traditional fossil energy forces the supply and demand of resources to turn to renewable energy such as wind energy, solar energy and tidal energy [1,2,3,4,5,6]. However, the intermittent nature of renewable energy cannot effectively sustain energy supply. In view of this, the development of energy storage devices with practical value and sustainable energy storage/conversion is the best way to solve the aforementioned problems. Supercapacitors, due to their low cost, fast charging and discharging, high power density, long cycle life and maintenance-free operation, are a new type of energy storage device coupled with batteries and traditional capacitors in portable and wearable equipment, and have great application prospects [7,8,9,10,11,12,13,14]. Electrode material is the key factor in determining the performance of supercapacitors. Among various electrode materials, carbon materials have been widely studied due to their low cost, large specific surface area, long cycle stability and simple preparation process [15,16,17,18,19,20,21,22].

However, when carbon materials are used as electrode materials for supercapacitors, the low effective utilization of their specific surface area results in many micropores that cannot effectively form an electric double layer. Therefore, optimizing the pore structure of carbon materials and increasing the specific surface area for effectively forming double electric layers will greatly improve the energy storage performance of electrode materials. As a two-dimensional layered nanostructure, graphene is widely regarded as the most ideal and promising electrode material for supercapacitors due to its high theoretical specific surface area (2630 m^2^ g^−1^), excellent electrical conductivity, excellent structural stability and other unique advantages [23,24,25]. However, the surface of pure graphene is inert and prone to irreversible aggregation, which greatly affects its electrochemical performance. Therefore, the functionalization of graphene and the synthesis of graphene-based composite materials have greatly improved the energy storage performance of composite electrode materials through the synergistic enhancement between nanomaterials [26,27,28,29,30,31].

Carbon nanotubes (CNTs), due to their unique hollow structure, excellent conductivity, interwoven network distribution of microstructure and good frequency response characteristics, have become an ideal candidate electrode material for high-power supercapacitors [32,33]. Unfortunately, the specific capacitance of CNTs is very small because CNTs usually have a low specific surface area (generally <200 m^2^ g^−1^) [34]. Therefore, the key to the commercial application of CNTs is to solve the problem of low specific volume. Surprisingly, the effective combination of CNTs and graphene can significantly enhance their electrochemical energy storage performance, which is mainly attributed to the following reasons. On the one hand, CNT can not only effectively reduce the irreversible aggregation of graphene and increase the space between graphene sheets, but also build a stable network with graphene, form a good electronic transmission channel, and greatly reduce the internal resistance of the electrode. On the other hand, the surface of graphene can get better release, greatly improve the effective utilization of specific surface area, and obtain a higher specific capacitance [35,36,37]. A functionalized graphene nanosheet/carbon nanotube network (G/CNTs-200) through chemical oxidation followed by low temperature treatment reduction is reported [34]. The G/CNTs-200 electrode exhibits a specific capacitance of 202 F g^−1^ at 0.5 A g^−1^ in 6 M KOH aqueous electrolyte, and a symmetric device based on G/CNTs-200 electrodes delivers an energy density of 11.7 Wh kg^−1^ and excellent electrochemical stability in 1 M Na_2_SO_4_ aqueous electrolyte, showing an outstanding electrochemical performance. The electrode of CNT-graphene fabricated via atmospheric pressure chemical vapor deposition (APCVD) showed a specific capacitance of 653.7 μF cm^−2^ at 10 mV s^−1^ and still delivered a high specific capacitance of 490.3 μF cm^−2^ at 300 mV s^−1^, demonstrating a great potential for high-performance supercapacitors [38]. Although some achievements have been made in carbon electrode research, it is still a great challenge to find and design more ideal electrode materials to improve the performance of electrochemical capacitors and ensure they meet market demands.

Herein, we successfully synthesized rGO-CNT nanocomposites via a one-step hydrothermal method with facile, low-cost, high efficiency, and large-scale preparation. Benefiting from the synergistic enhancement effect between the two electrode materials and the construction of a unique hierarchical porous network nanostructure that can make full use of the conductive network structure of CNTs and the high specific surface area of rGO, the optimum rGO-CNT_30_ nanohybrid electrode demonstrates good charge transfer conductance, high specific capacitance, and excellent rate capability. Most importantly, the assembled symmetrical supercapacitor system (SSS) using a rGO-CNT_30_ electrode possesses a high energy density and an extraordinary cycle stability, which has great application potential as an electrode material for next-generation electrochemical capacitors. 

## 2. Experimental Section

### 2.1. Materials Synthesis

Graphene oxide (GO) is prepared from natural flake graphite powder by a modified Hummer’s method (Electronic Supplementary Materials). In a typical hydrothermal procedure, 100 mg GO is dispersed in 100 mL deionized water and sonicated for 1 h at room temperature. Then 10 mg of carbon nanotubes (CNTs) are added to the above solution, and ultrasonic stirring was continued for 2 h. Next, the mixture is stirred magnetically for 3 h to form a homogeneous solution. Afterwards, the mixture is transferred to Teflon-lined stainless steel autoclave for a hydrothermal process at 180 °C for 10 h. After cooling to room temperature, the reaction product is collected by centrifugation and dried in vacuum at 65 °C for 18 h. The final product is denoted as rGO-CNT_10_. For comparison, the nanomaterials with various weights of CNTs (0 mg, 20 mg, 30 mg, and 50 mg) are synthesized using the same experimental method and denoted as rGO, rGO-CNT_20_, rGO-CNT_30_ and rGO-CNT_50_, respectively.

### 2.2. Characterization

Crystal phase of samples is characterized by X-ray powder diffraction (XRD) using a Bruker D8 Advance diffractometer (Bruker AXS Co., Ltd., German) with Cu Kα radiation (λ = 1.5417 Å) at 40 kV and 40 mA. Raman spectrum is obtained using a Renishaw inVia Raman microscope (Renishaw, Gloucestershire, London, UK) employing a 633 nm laser excitation (25% laser power). The morphology and microstructure of the synthesized samples are characterized by field-emission scanning electron microscopy (FESEM, Quanta 250 FEG) (FEI Company, Hillsboro, Oregon, U.S.). The pore size distribution and specific surface area of the as-synthesized nanocomposites are calculated from Brunauer–Emmett–Teller (BET) and Barrett–Joyner–Halenda (BJH) calculation model by using a Micromeritics ASAP-2020 (McMuritik Instrument Co., Ltd., U.S.) at 77 K after being degassed at 150 ℃ for 6 h.

### 2.3. Electrochemical Measurements

All electrochemical measurements are evaluated using a CHI 760E electrochemical work station (Shanghai Chenhua Instrument Co., Ltd., China) by both three-electrode and two-electrode devices. Working electrodes are prepared using a mixture of as-prepared active materials (80 wt%), PTFE (10 wt%) and conductive carbon black (10 wt%), and the isopropyl alcohol is used as the solvent. After mixing evenly, the slurry is coated and pressed onto nickel foam with an area of about 1 cm^2^, and dried under vacuum at 75 °C for overnight. The mass loading of the active materials is about 1.7 mg cm^−2^ for all prepared electrodes. The three-electrode system is comprised of of platinum foil and saturated calomel electrodes as the counter and reference electrodes. The prepared electrode is pre-soaked in aqueous electrolyte for about 5 h to provide sufficient contact for the electrolyte and working electrode before the electrochemical behavior test. The gravimetric capacitance is evaluated from the GCD curves and CV curves according to the following Equations (1) and (2), respectively:

(1) $$C=\frac{I\Delta t}{m\Delta V}$$

(2) $$C=\frac{\int I\left(V\right)dV}{mv\Delta V}$$

where *C* is the specific capacitance (F g^−1^), *I* is the response current (A), *m* is loading mass of electroactive materials (g), Δ*t* is the discharge time (s), Δ*V* and *v* are the potential window (V) and the scan rate (V s^−1^), respectively.

The alternating current (AC) complex capacitance C*(ω) = *C*′ (ω)—j*C*″ (ω) is calculated from the impedance data, and the *C*′ (ω) and *C*″ (ω) are calculated according to the following equations [39]:

(3) $$C^{\prime }\left(\omega \right)=\frac{-Z^{\prime \prime }\left(\omega \right)}{\omega {\left|Z\left(\omega \right)\right|}^{2}}$$

(4) $$C^{\prime \prime }\left(\omega \right)=\frac{-Z^{\prime }\left(\omega \right)}{\omega {\left|Z\left(\omega \right)\right|}^{2}}$$

where *C′ (ω)* and *C″ (ω)* respectively represent the real part and the imaginary part related to the losses in the charge storage process leading to an energy dissipation. The *ω* is the angular frequency and it is given by ω = 2πf, and *f* represents the frequency. *J* is the concept of complex numbers, and j is the symbol of imaginary number.

Hereupon, the symmetric supercapacitor system (SSS) is assembled by using the rGO-CNT_30_ electrode and soaked in 1 M Na_2_SO_4_ aqueous solution for 5 h before the test. The gravimetric specific capacitance, energy density (*E*) and power density (*P*) of the assembled rGO-CNT_30_//rGO-CNT_30_ SSS are calculated based on the following equations:

(5) $$C=\frac{I\Delta t}{M\Delta V}$$

(6) $$E=\frac{1}{2\cdot 3.6}C{\left(\Delta V\right)}^{2}$$

(7) $$P=\frac{3600E}{\Delta t}$$

where *I* is the discharge current (A), Δ*t* is the discharge time (s), Δ*V* is the potential window (V), and *M* is the total mass of both electrodes (g).

## 3. Results and Discussion

**Figure 1:**
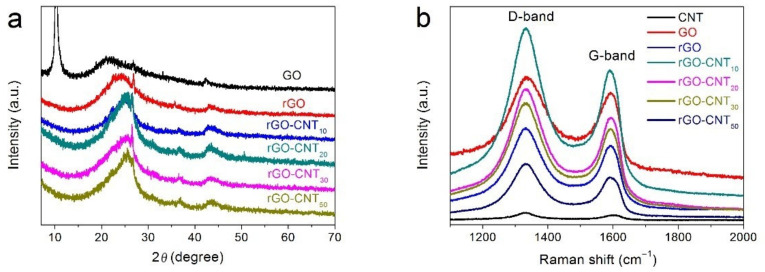
(**a**) XRD patterns of GO, rGO, and rGO-CNT samples. (**b**) Raman spectra of CNT, GO, rGO, and rGO-CNT samples.

The crystal structure and phase of the as-prepared samples are characterized by XRD, as illustrated in Figure 1a. It can be observed that the diffraction peak of the GO sample mainly consists of a sharp diffraction peak at 2*θ* = 10.4°, a broad bulge peak at around 2*θ* = 22.6° and a weak peak located at about 2*θ* = 42.3°. After hydrothermal reduction, the sharp peaks of rGO and rGO-CNT samples at 2*θ* = 10.4° disappeared, and only characteristic peaks near 2*θ* = 26.3° and 2*θ* = 42.8° were found, indicating that GO was successfully reduced to rGO. In addition, the crystallinity of the rGO-CNT composite material changes slightly with the weight of the added CNTs. The results show that the crystallinity of the rGO-CNT30 sample is slightly higher than that of other rGO-CNT composite materials. Raman spectroscopy was used to further characterize the structural information of CNT, GO, rGO and rGO-CNT samples. As shown in Figure 1b, all samples showed two main characteristic peaks in the D band (~1331 cm^−1^) and G band (~1592 cm^−1^). The peak of the D band is generally considered as the disordered vibration peak of graphene (carbon) materials, reflecting the structural defects in graphene materials. The peak of the G-band is caused by the E_2g_ phonon in-plane vibration of sp^2^ hybridized carbon atoms [40,41,42]. The intensity ratio of I_D_/I_G_ is an important parameter to evaluate the degree of structural disorder and lattice defects of carbonaceous materials, especially for CNT/graphene-based materials. As summarized in Table 1, the increase of the I_D_/I_G_ value for rGO sample shows that more disordered structures and defects are produced after hydrothermal reduction at high temperature. Furthermore, based on the high intensity ratio of I_D_/I_G_ of CNT material, it can be seen that the overall *I_D_*/*I_G_* value of the rGO-CNT composite gradually increases with the increase of the CNT material reaction amount, indicating that more defects are formed and will provide more channels and active sites for electron transfer [43]. In addition, as a whole, the peak position of the rGO-CNT composite shows a slight blue shift, which also proves that the interaction between the CNT and rGO materials causes an increase of defects [44].

**Figure 2:**
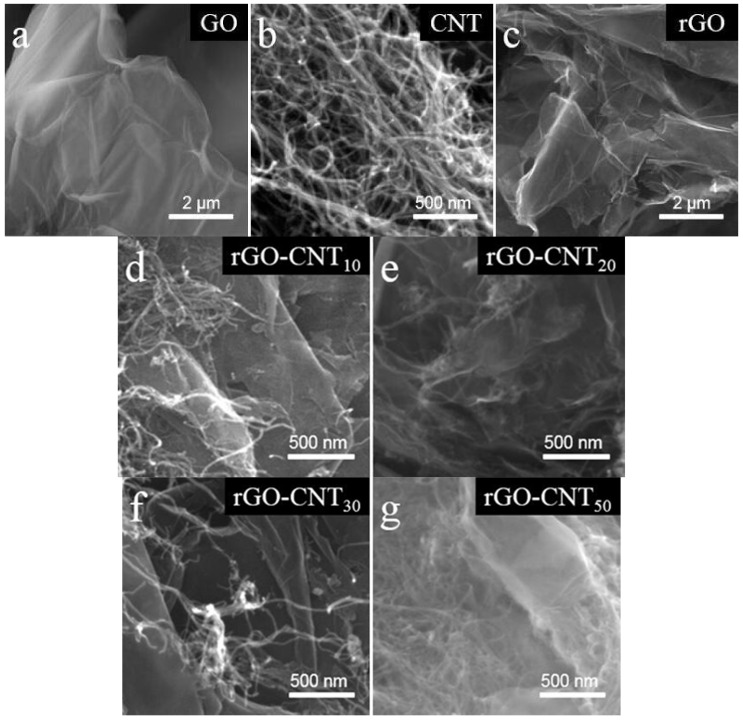
SEM images of (**a**) GO, (**b**) CNT, (**c**) rGO, (**d**) rGO-CNT_10_, (**e**) rGO-CNT_20_, (**f**) rGO-CNT_30_ and (**g**) rGO-CNT_50_ samples.

The surface morphology of CNT, GO, rGO, and rGO-CNT samples are investigated by field emission scanning electron microscope (FESEM). Through careful comparison of Figure 2a,c, it can be found that after hydrothermal reduction, the obtained rGO shows a more fluffy and porous lamellar-nanostructure, and the serious agglomeration phenomenon of GO is obviously improved. Figure 2b shows the SEM image of the CNT, where CNTs are cross-linked and entangled with each other, and the diameter of single nanotube is about 10 nm. Similar to graphene, some carbon nanotubes also have a certain degree of agglomeration. The SEM images of the rGO-CNT composites synthesized with different amount of CNTs involved in the reaction are shown in Figure 2d–g. It can be clearly seen that some CNTs have a certain degree of aggregation, which is attributed to their easy aggregation characteristics. However, there are also a large number of CNTs that can be well dispersed in the nanosheet layers of rGO. By further comparison, it can be found that the reactant CNTs are too small (Figure 2d), the rGO nanosheets are not better dispersed, while the reactant CNTs are too high (Figure 2g), and the CNTs that easily aggregate are not well dispersed. Therefore, an appropriate amount of reactant CNT participating in the reaction (Figure 2e,f) can more effectively reduce the agglomeration and stacking of CNTs and rGO with easy agglomeration characteristics, so as to obtain composites with more abundant porous and stable nanostructures.

**Figure 3:**
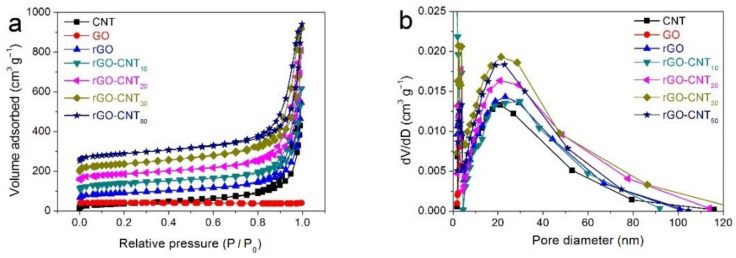
(**a**) Nitrogen adsorption-desorption isotherms, and (**b**) the pore size distribution curves of CNT, GO, rGO, rGO-CNT_10_, rGO-CNT_20_, rGO-CNT_30_, and rGO-CNT_50_ samples.

The specific surface area, pore structure, and distribution of the prepared materials are detected by nitrogen adsorption-desorption isotherm. As illustrated in Figure 3a, the isotherms of all samples except the GO material are classified as a typical type-IV isotherm with a hysteresis loop at *P*/*P_0_* of about 0.8, suggesting the existence of substantial mesopore structures and a small amount of macroporous structures. Further, the isotherm of the GO material, which is almost parallel to the horizontal axis, shows its very poor pore structure. The corresponding BJH pore size distribution curves of CNT, GO, rGO, rGO-CNT_10_, rGO-CNT_20_, rGO-CNT_30_, and rGO-CNT_50_ samples are displayed in Figure 3b. It can be observed that these as-synthesized samples (except GO) are mainly composed of a vast majority of mesopores with a diameter of about 10–50 nm and a very small amount of macropores and micropores. In composite materials with a porous structure, micropores can provide more active sites, mesopores are conducive to full penetration of electrolytes and full contact of ions with electrode materials, and macropores can act as electrolyte ion buffer pools, so this composite material with a hierarchical porous structure is conducive to high-efficiency ion transport and electrochemical energy storage efficiency and ensures excellent cycle performance. Additionally, the mesopores can provide a larger specific surface area for ion transport/charge storage, thus enhancing the double-layer capacitance and pseudocapacitance [44,45,46,47]. The visual data of specific surface areas, pore diameters and pore volumes of the prepared samples are summarized in Table S1. It can be observed that the specific surface area and porosity of GO are greatly improved after reduction, which means that the agglomeration of graphene is obviously improved. In addition, the specific surface area of the composite is also higher than that of a single electrode material. With the increase of CNT content, the specific surface area of the rGO-CNT material is also gradually increased. Additionally, the rGO-CNT_30_ composite exhibits the largest specific surface area of 204.12 m^2^ g^−^^1^. However, the excessive CNTs in composite rGO-CNT_50_ will lead to agglomeration in a larger area, which will reduce the specific surface area and porosity of the composites. These can also be verified by the change of the total volume of the rGO-CNT composites. In addition, compared with CNT and rGO materials, we can see that the average pore diameter of the composite increases slightly. Since the addition of carbon nanotubes provides a more bulky structure for the composite material, this provides a better structural basis and more reduction sites for the reduction of graphene oxide, which should lead to a further improvement in the pore structure of the composite material.

**Figure 4:**
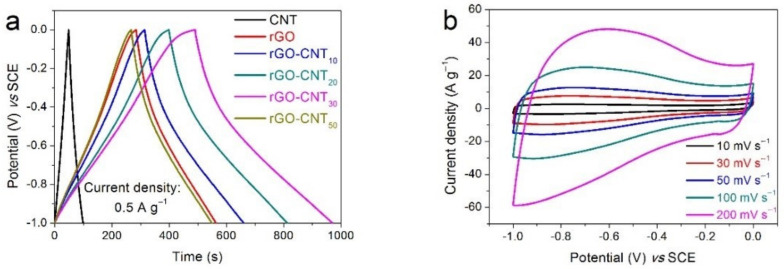

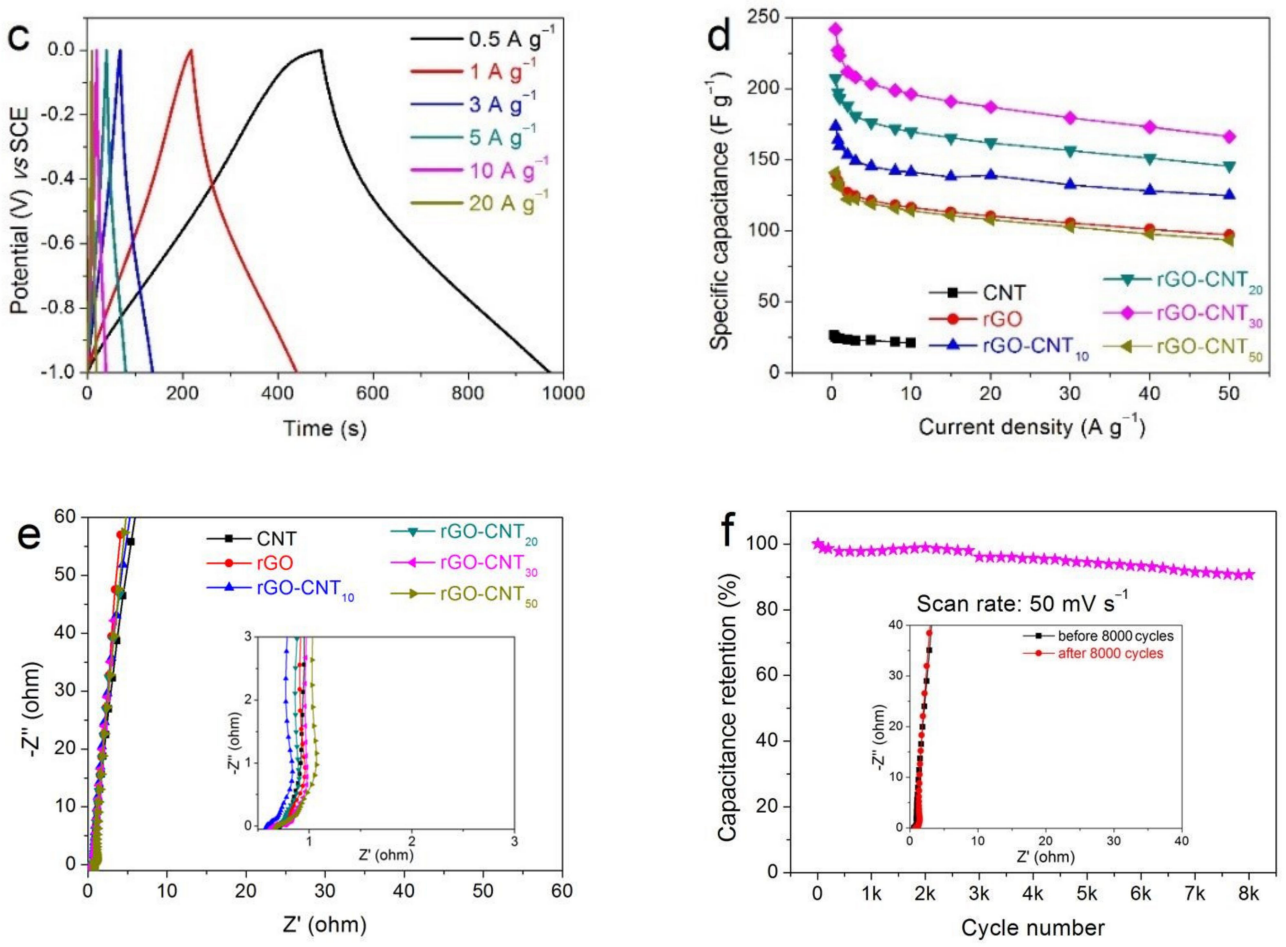
Electrochemical performances of all electrodes in 2 M KOH electrolyte: (**a**) GCD curves at a current density of 0.5 A g^−1^ for CNT, rGO, and rGO-CNT electrodes, (**b**) CV curves at various scan rate, and (**c**) GCD curves at different current densities for rGO-CNT_30_ electrode, (**d**) Specific capacitance *vs* current density, and (**e**) Nyquist plots for CNT, rGO, and rGO-CNT electrodes, (**f**) Long-term cycling performances of the rGO-CNT_30_ electrode for 8000 cycles (the inset shows the Nyquist plots before and after 8000 cycles).

In order to have a better understanding of the electrochemical performances of the synthesized nanocomposites, we investigated and compared the electrochemical performances of the prepared electrodes in different electrolytes. The electrochemical performances of the rGO-CNT electrodes in 2M KOH electrolyte are displayed in Figure 4. As shown in Figure 4a, the specific capacitances of CNT, rGO, rGO-CNT10, rGO-CNT20, rGO-CNT30 and rGO-CNT50 electrodes at 0.5 A g^−1^ are 26.4, 139.75, 173.30, 207.07, 241.62, and 141.1 F g^−1^, respectively. The results show that rGO-CNT_30_ electrode has the best capacitance behavior, which can also be verified from the CV test curve (Figure S1a,b). The CV curves of the rGO-CNT_30_ electrode at various scan rates from 10 to 200 mV s^−1^ are shown in Figure 4b. Benefiting from the pseudocapacitance contribution brought by a large number of oxyfunctional groups in the composite material, the CV curves of the carbon electrode exhibit a distorted rectangular shape with a wide redox band, indicating the presence of double layer capacitance and pseudocapacitance during electrochemical energy storage. Furthermore, the fast response of current to voltage reversal at each end potential shows a good, high-speed reversible electrochemical behavior [48]. The GCD curves of the rGO-CNT_30_ electrode at different current densities are also presented in Figure 4c. The non-linear GCD curves further indicate the existence of pseudocapacitance, which mainly comes from unreduced functional groups of rGO. In addition, the almost non-existent IR drop even at high current density indicates the low resistance and high conductivity of the rGO-CNT_30_ electrode. The specific capacitances of CNT, rGO and rGO-CNT electrodes calculated from their corresponding GCD curves at different current densities are shown in Figure 4d. As seen, the specific capacitance of the rGO-CNT_30_ electrode is the largest among the prepared electrodes in the entire current density range, indicating excellent capacitive behavior. Furthermore, the rGO-CNT_30_ electrode still maintains a specific capacitance of 166.63 F g^−1^ at a current density of up to 50 A g^−1^, demonstrating a comparatively good rate capability. 

The electrochemical impedance spectroscopy (EIS) of these electrodes is carried out over a frequency range of 100 kHz to 0.01 Hz to investigate the ion transport behavior and electrical resistance. As shown in Figure 4e, a semicircle arc in the high-frequency region and a straight line in the low-frequency region can be observed in the Nyquist plots of all electrodes. The intercept at high frequency with real axis is the electrolyte resistance (Re), and the semicircular arc radius at high-medium frequency represents the charge transfer resistance (*Rct*) [49]. It can be observed from Figure 4e that all electrodes exhibit a smaller Rct, demonstrating fast kinetics. Moreover, the inclined slope at low frequency represents the Warburg impedance, and an approximate vertical line in this region indicates a good capacitive-type behavior, which suggests that it is relatively easier for ions diffusion at the electrode/electrolyte interface. The frequency dependence of complex C*(ω) is represented by a relaxation behavior that reduces the capacitive response above some frequencies. The *C′* (ω) and *C″* (ω) values calculated from the impedance data are shown in Figure S1c,d respectively. It can be observed that the rGO-CNT_30_ and rGO-CNT_20_ electrodes exhibit a higher capacitance at low frequencies in Figure S1c, which is consistent with the test results of CV and GCD. The *C″* versus frequency dependencies show a maximum at 5623, 681.3, 1000, 562.3, 464.2 and 681.3 mHz for CNT, rGO, rGO-CNT_10_, rGO-CNT_20_, rGO-CNT_30_ and rGO-CNT_50_ electrodes, respectively. These electrodes still have outstanding electrochemical properties at high frequencies, indicating a great potential for practical applications in high-power energy storage devices [42,50].

The long-term cycling stability is another important requirement for electrode materials. Figure 4f displays the cycling stability of rGO-CNT_30_ electrode by repeating the CV test at a scan rate of 50 mV s^−1^ for 8000 cycles. It can be found that the rGO-CNT_30_ electrode still retained 90.66% of initial capacitance after 8000 cycles, indicating an outstanding cycling stability and a very high degree of reversibility in repeated scanning cycle. The inset in Figure 4f shows the comparison of the Nyquist plots before and after 8000 cycles. It can be observed that after long-term cycling, the Re and Rct increase slightly, but the Warburg impedance hardly increases, which is mainly due to the diffusion process without much change. In general, the collapse of the microstructure of the carbon material during the long-period charge and discharge cycle can easily damage the cycle stability of the electrode. The excellent cycle stability of rGO-CNT_30_ indicates that it has good structural stability.

**Figure 5:**
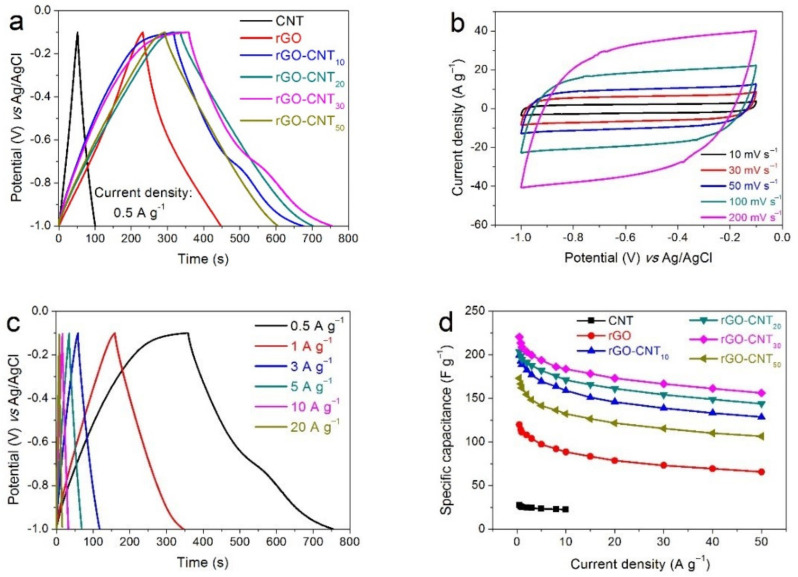

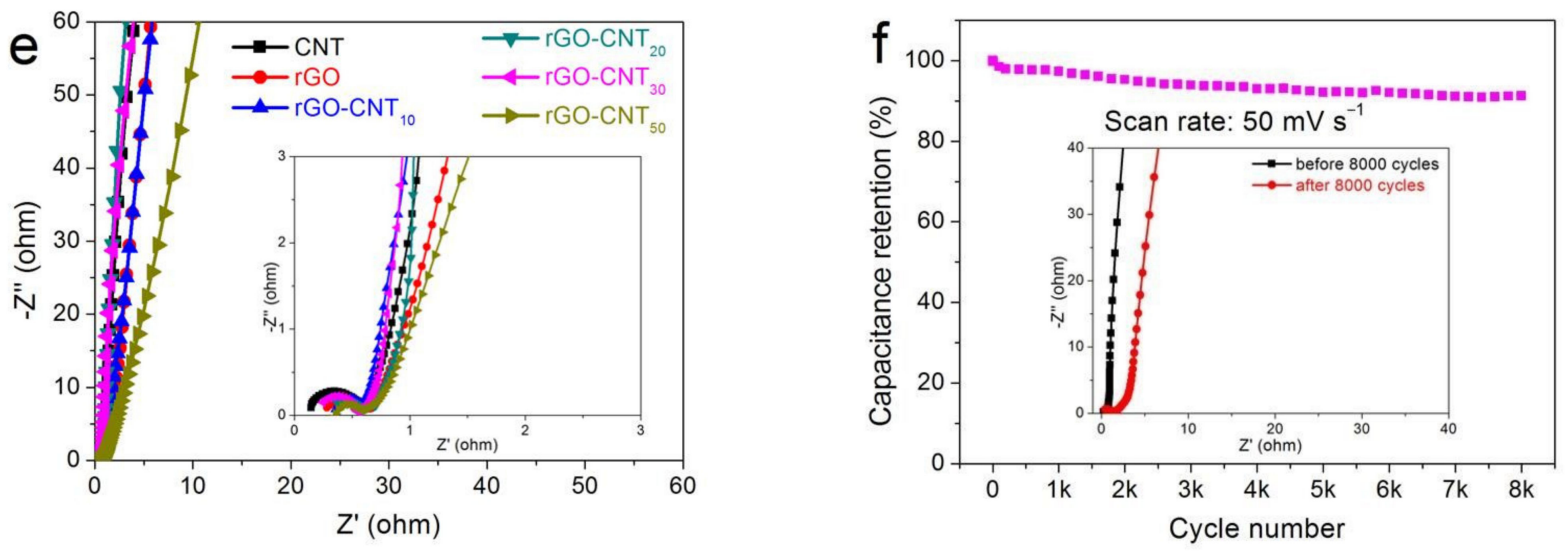
Electrochemical performances of all electrodes in 1 M Na_2_SO_4_ electrolyte: (**a**) GCD curves at a current density of 0.5 A g^−1^ for the CNT, rGO, and rGO-CNT electrodes, (**b**) CV curves at various scan rate, and (**c**) GCD curves at different current densities for the rGO-CNT_30_ electrode, (**d**) Specific capacitance *vs* current density, and (**e**) Nyquist plots for the CNT, rGO, and rGO-CNT electrodes, (**f**) Long-term cycling performances of the rGO-CNT_30_ electrode for 8000 cycles (the inset shows the Nyquist plots before and after 8000 cycles).

Figure 5 displays the electrochemical performances of the rGO-CNT nanocomposite electrodes in 1 M Na_2_SO_4_ electrolyte. According to its GCD curve (Figure 5a), the rGO-CNT_30_ electrode has the highest specific capacitance of 220.33 F g^−1^ at 0.5 A g^−1^, which is higher than that of the CNT (27.12 F g^−1^), rGO (119.83 F g^−1^), rGO-CNT_10_ (199.23 F g^−1^), rGO-CNT_20_ (203.52 F g^−1^), and rGO-CNT_50_ (173.13 F g^−1^) electrodes. In addition, the non-linear GCD curves in rGO and rGO-CNT electrodes indicate the existence of pseudocapacitance. The CV curves of the rGO-CNT_30_ electrode displays similar quasi-rectangular shapes at various scan rates (Figure 5b), indicating the capacitance produced in charge-discharge process is mainly electric double layer capacitance. Figure 5c presents the GCD curves of the rGO-CNT_30_ electrode at different current densities from 0.5 to 20 A g^−1^. It can be seen that with the increase of current density, the electrode capacitance shows more and more obvious characteristics of electric double layer capacitance, which also means that the proportion of electric double layer capacitance in the generated capacitance increases gradually. In addition, the ultra-low IR drop shows that the rGO-CNT_30_ electrode has excellent conductivity. The specific capacitances *vs* current densities of the CNT, rGO and rGO-CNT electrodes are displayed in Figure 5d. It can be seen that compared with CNT and rGO electrodes, the electrochemical performance of rthe GO-CNT composite electrodes is significantly enhanced at various current densities. The performance of the rGO-CNT_30_ electrode is particularly prominent. Moreover, when the current density increases to 50 A g^−1^, the rGO-CNT_30_ electrode still exhibits a specific capacitance of 156.21 F g^−1^, showing an excellent rate capability.

The Nyquist plots of the CNT, rGO, and rGO-CNT electrodes are presented in Figure 5e. It can be seen that almost all curves are composed of a semicircle arc in the high-frequency region and an approximate straight line in the low frequency region. A very small intercept between the Nyquist plots in the high-frequency region and the *x*-axis indicates that these electrodes have a very small electrolyte resistance (*Re*) in this test system. Additionally, we can also know that these electrode materials have a small charge transfer resistance (*Rct*), which can be reflected from the small diameter of the semicircle arc in the high frequency area of these Nyquist plots. The slope of the approximate straight line in the low-frequency region in Nyquist plots reflects the Warburg impedance of the electrode in the test system, and the high slope means that the electrode has better and more ideal capacitive behavior. By careful comparison, it can be seen from Figure 5e that among these electrodes, rGO-CNT_20_ and rGO-CNT_30_ electrodes have smaller Warburg resistance, which means they have better capacitive behavior and better ion diffusion kinetics properties. In addition, we speculate that the poor capacitive behavior of the rGO-CNT_50_ electrode should be mainly attributed to the severe agglomeration of composite materials caused by excessive CNT material and the formation of poor pore nanostructure. 

The cycling stability of the rGO-CNT_30_ electrode presented in Figure 5f shows a capacitance retention of 91.23% after 8000 cycles at 50 mV s^−1^, demonstrating an excellent long-term cycling stability. Comparing the Nyquist plots before and after 8000 cycles (the inset in Figure 5f), it can be seen that the Rct and Warburg impedance after 8000 cycles only slightly increase, which indicates that shows the excellent structural stability and cyclic stability of the rGO-CNT_30_ composite electrode.

By comparing the electrochemical properties of the electrode materials in 2 M KOH and 1 M Na_2_SO_4_ aqueous electrolyte, it can be seen that the rGO-CNT composite electrodes exhibit excellent electrochemical properties in two electrolytes, but with slight differences. Taking the rGO-CNT_30_ electrode material with optimal performance as an example, it can be found that although the rGO-CNT_30_ electrode has a lower specific capacitance (220.33 F g^−1^ < 241.62 F g^−1^) in the Na_2_SO_4_ electrolyte, it shows better rate capability (70.90% > 68.76% (0.5–50 A g^−1^)) and cycling stability (91.23% > 90.66%). This indicates that different ions involved in the charge-discharge reaction will have different effects on the electrochemical properties of the electrode material. The rGO-CNT_30_ electrode possesses a high specific capacitance, excellent rate capability, exceptional conductivity and outstanding long-term cycling stability, so it is very worth looking forward to this material being assembled into a supercapacitor device to further explore its practical performance. 

**Figure 6:**
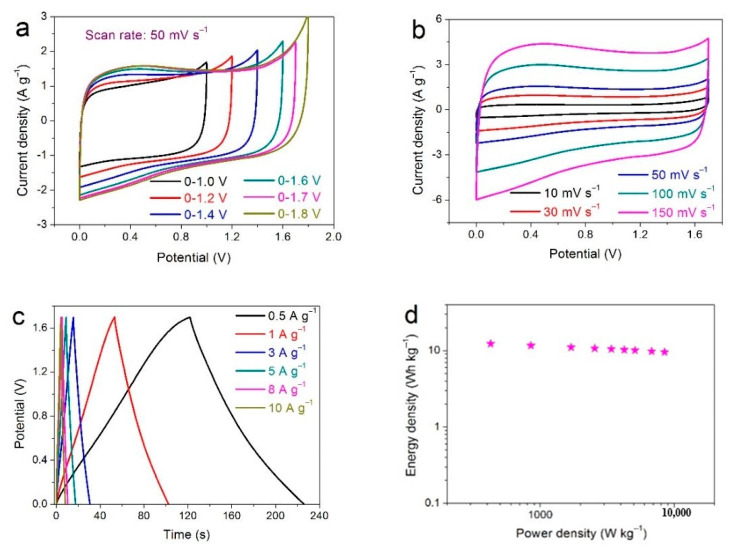

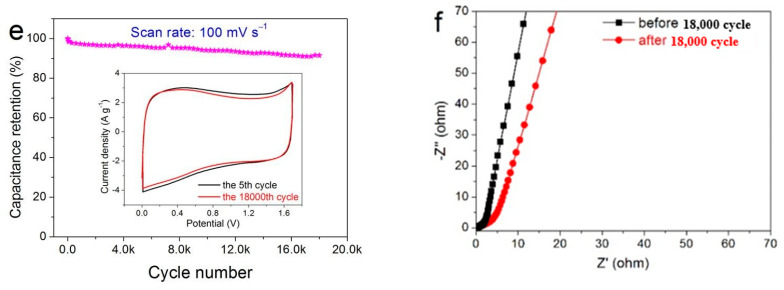
CV curves of rGO-CNT_30_//rGO-CNT_30_ SSS (**a**) at different potential windows from 0−1.0 V to 0−1.8 V with a scan rate of 50 mV s^−1^, and (**b**) at various scan rates. (**c**) GCD curves at different current densities, and (**d**) Ragone plots of rGO-CNT_30_//rGO-CNT_30_ SSS. (**e**) Capacitance retention of the rGO-CNT_30_//rGO-CNT_30_ SSS over 18,000 cycles at a scan rate of 100 mV s^−1^ (Insert shows the CV curves of the 5th cycle and the 18000th cycle), (**f**) the Nyquist plots before and after 18,000 cycles.

A symmetric supercapacitor system (SSS) is assembled using the rGO-CNT_30_ electrode as positive electrode and negative electrode, a cellulose paper as the separator, and 1 M Na_2_SO_4_ aqueous solution as the electrolyte. The CV curves of different potential windows under 50 mV s^−1^ are shown in Figure 6a. After comparison, 0−1.7 V is the best working potential window under the precursor that avoids polarization phenomenon. Figure 6b represents CV curves of rGO-CNT_30_//rGO-CNT_30_ SSS at various scan rates from 10 to 150 mV s^−1^. The CV curve of quasi-rectangle shape indicates a characteristic of a relatively ideal capacitor behavior, consisting of the double layer behavior and pseudocapacitance behavior in the reaction process. In addition, the CV curve of the SSS still keeps the quasi-rectangle shape even if the scanning rate increases to 800 mV s^−1^ (Figure S2), showing distinguished cycle reversibility and remarkable rate capability. The GCD curves of rGO-CNT_30_//rGO-CNT_30_ SSS at different current densities from 0.5 to 10 A g^−1^ are displayed in Figure 6c. All the curves are quasi-linear with very small IR drop and show a typical triangular shape, which further demonstrates an excellent rate capability and an ideal electrochemical capacitive behavior [48,51]. Calculated according to the GCD curves, the Ragone plots of rGO-CNT_30_//rGO-CNT_30_ SSS are displayed in Figure 6d, which is an important parameter to determine the overall electrochemical performance of electrode materials for supercapacitors. It can be observed that the SSS obtains a high energy density of 12.29 W h kg^−1^ (425 W kg^−1^) and a power density up to 8500 W kg^−1^ (9.56 W h kg^−1^), which is superior to other previously reported carbon-based symmetric supercapacitors [34,48,51,52,53,54,55]. 

Long-term cycle stability of the rGO-CNT_30_//rGO-CNT_30_ SSS is presented in Figure 6e. In our experiment, the repeating CV test is used to quantitatively describe cycling performance for 18,000 cycles at a scan rate of 100 mV s^−1^. The specific capacitance of the SSS still remains 91.42% of its initial capacitance over for 18,000 cycles, showing excellent cycle life. The insert in Figure 6e shows the comparison between the 5th and 18000th CV curves. It can be seen that the CV curve of the 18000th cycle retains the original shape, except that the slightly polarization and the enclosed area are slightly reduced, which fully shows the ultra-long cycle stability of the rGO-CNT_30_//rGO-CNT_30_ SSS. Figure 6f delivers the Nyquist plots before and after 18,000 cycles. Through careful observation, it can be found that after 18,000 cycles of charging and discharging, the diameter of semicircle arc in the middle-high frequency region slightly increases and the slope of straight line in the low frequency region slightly decreases, indicating that the Rct and Warburg impedance of the SSS slightly increase. This is the main reason for capacitance decay. Overall, the comprehensive resistance of the device is still very small, which is mainly due to the synergistic effect of composite materials to enhance the structural stability, thus obtaining excellent ultra-long cycle stability. The rGO-CNT_30_ composite with excellent electrochemical performances is superior to the carbon electrode supercapacitor reported previously (Table 2) [56,57,58,59,60,61].

## 4. Conclusions

In summary, free-standing rGO/CNT composites have been synthesized by a facile hydrothermal method. The introduction of CNTs can form an interweaved network structure to enhance the conductivity of rGO and build a stable supporting structure, and the rGO has an ultra-high theoretical specific surface area. The effective combination and synergistic enhancement of the two electrode materials can greatly reduce the inherent characteristics of easy agglomeration, thus effectively increasing the electrochemical energy storage performance of the composite electrode materials. Benefiting from their synergistic enhancement, the optimal rGO-CNT_30_ electrode exhibits high specific capacitance, excellent rate capability, exceptional conductivity and outstanding long-term cycling stability. Furthermore, the assembled rGO-CNT_30_//rGO-CNT_30_ SSS delivers a high energy density of 12.29 W h kg^−1^ (425 W kg^−1^) and a high power density up to 8500 W kg^−1^ (9.56 W h kg^−1^), as well as an excellent ultra-long cycle stability (91.42%). This work provides a promising self-supporting electrode material for supercapacitor application, and enriches new carbon-based energy storage devices.

## Figures and Tables

**Table 1:** Raman spectroscopy parameters of the CNT, GO, rGO, rGO-CNT_10_, rGO-CNT_20_, rGO-CNT_30_, and rGO-CNT_50_ samples.

Sample	D-Band (cm^−1^)	G-Band (cm^−1^)	I_D_/I_G_
CNT	1329	1592.4	1.25
GO	1336.2	1592.8	1.12
rGO	1331.1	1593.4	1.23
rGO-CNT_10_	1333.3	1589.3	1.27
rGO-CNT_20_	1332.2	1594.5	1.26
rGO-CNT_30_	1334.4	1592.4	1.27
rGO-CNT_50_	1330.1	1594.5	1.29

**Table 2:** Comparison of electrochemical performances for the similar carbon-based composite electrodes in previous literature.

Samples	Specific Capacitance (F g^−1^)	Current Density/Scan Rate	Energy Density (W h kg^−1^) /Power Density (W kg^−1^)	Capacitance Retention (Cycle Numbers)	Ref.
G/GNTs-200	202	0.5 A g^−1^	11.7/50	102% (20,000)	[34]
Ti_3_C_2_/CNTs	134	1 A g^−1^	2.77/311	>100% (10,000)	[51]
PPy-RGO	255.7	0.2 A g^−1^	7.02/89	93% 1000	[56]
Ap-rGO	160	5 mV s^−1^	5.6/50	85% (5000)	[57]
Polyaniline/ Carbon Nanotube	359	1.56 mA cm^-2^	11.1/980	92% (10,000)	[58]
RGO/UCNTs/PANI	359.3	1 A g^−1^	7.4/189	80.5% (2000)	[59]
rGO/BiVO_4_	196	5 mV s^−1^	33.7/1140 and 15.33/8000	80.3% (2000)	[60]
MP-VOPO_4_@rGO	672	1 A g^−1^	26.3/250 and 10.1/5000	83.5% (5000)	[61]
rGO-CNT_30_	241.62	0.5 A g^−1^	12.29/425 and 9.56/8500	91.42% (18,000)	This work

## Data Availability

The data used to support the findings of this study are available from the corresponding author upon request.

## Supplementary Materials

The following are available online at https://www.mdpi.com/article/10.3390/nano11061420/s1, Figure S1: (a) CV curves, (b) Specific capacitance, (c) *C*′, and (d) *C*″ calculated from the impedance data vs frequency of CNT, rGO, rGO-CNT10, rGO-CNT20, rGO-CNT30, and rGO-CNT50 samples, Figure S2: CV curves of rGO-CNT30//rGO-CNT30 SSS at various scan rates from 200 to 800 mV s^−1^, Table S1: Textural parameters of the CNT, GO, rGO and rGO-CNTs samples.
