# Supplementary material for: Free-Standing rGO-CNT Nanocomposites with Excellent Rate Capability and Cycling Stability for Na2SO4 Aqueous Electrolyte Supercapacitors
Source: Nanomaterials (Basel). 2021 May 28;11(6):1420. doi: 10.3390/nano11061420 (PMC8229913; doi:10.3390/nano11061420)
Supplement: Supplementary file 1 [file nanomaterials-11-01420-s001.zip › nanomaterials-1172973-supplementary.pdf]

Supplementary Information

Free-standing rGO-CNT Nanocomposites with Excellent Rate Capability and Cycling Stability for Na_2SO_4 Aqueous Electrolyte Supercapacitors

1. Preparation of graphene oxide (GO)

In a modified Hummer's method, 1.5 g of natural powdered flake graphite is added into a beaker containing 50.7 mL of concentrated sulfuric acid and stirred magnetically in ice-water bath. 10 minutes later, 1.14 g of sodium nitrate is slowly added into it. And after two hours, add 3.0 g of potassium permanganate into it slowly and keep stirring continuously. Three days later, 150 ml of sulfuric acid (5%) and 4.5 mL of H_2O_2 (30%) are added into the above solution in turn and slowly under continuous magnetic stirring, which we can observe that the color of solution gradually turning bright yellow. After standing for half a day, the yellow mixture is centrifuged several times with dilute hydrochloric acid (10%), then washed with plenty of DI water until nearly neutral (pH ~ 7). Finally, GO can be obtained by freeze-drying the obtained viscous solution after centrifugation for 48 hours.

2. Supplementary Figures

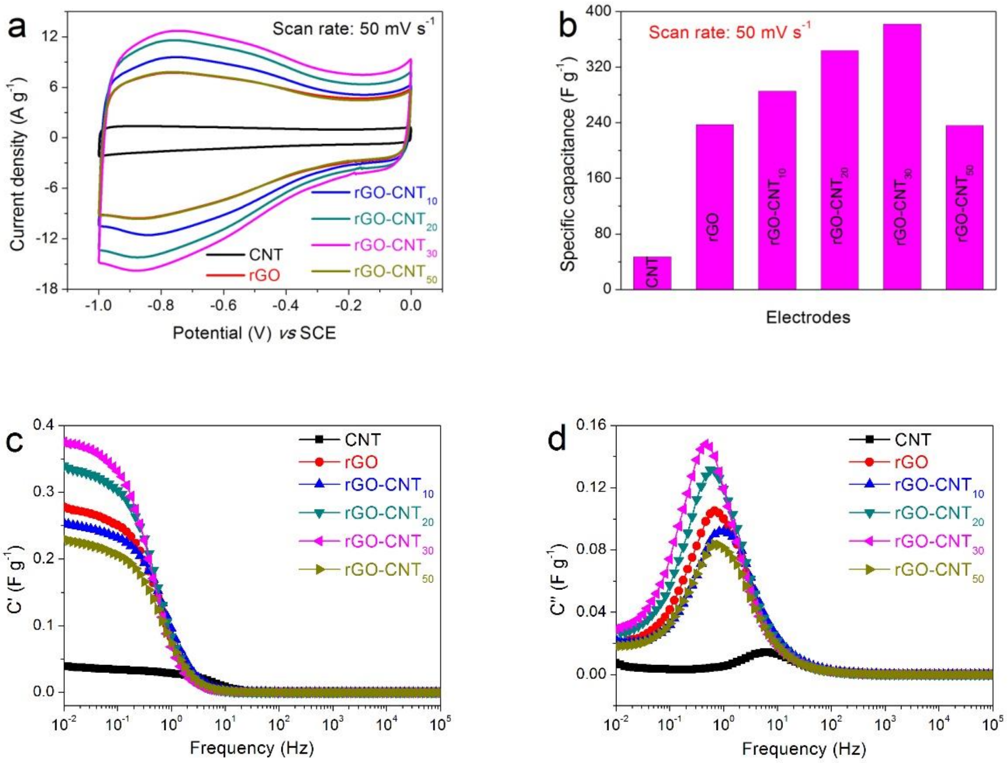

Figure S1. (**a**) CV curves, (**b**) Specific capacitance, (**c**) *C′*, and (**d**) *C″* calculated from the impedance data *vs* frequency of CNT, rGO, rGO-CNT_{10}, rGO-CNT_{20}, rGO-CNT_{30}, and rGO-CNT_{50} samples.

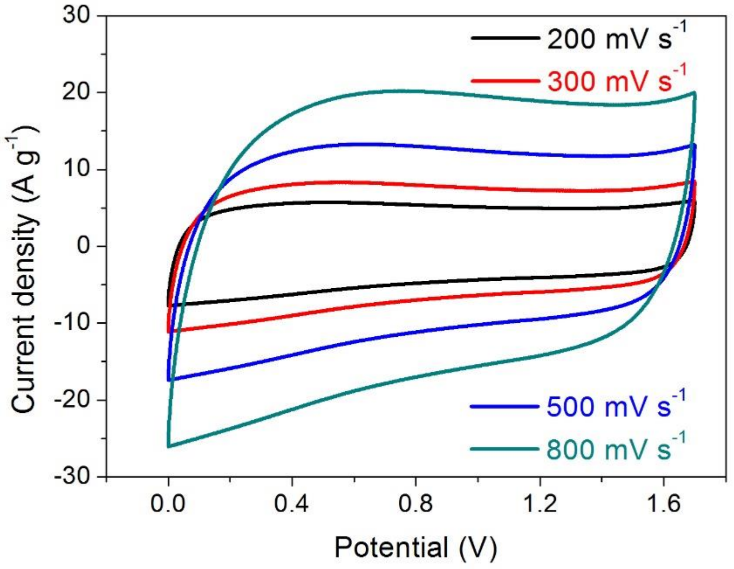

Figure S2. CV curves of rGO-CNT_{30}//rGO-CNT_{30} SSS at various scan rates from 200 to 800 mV s^{-1}.

Table S1. Textural parameters of the CNT, GO, rGO and rGO-CNTs samples

Sample	S_{BET} (m^2 g^{-1})	D_{DFT} (nm)	V (cm^3 g^{-1})
CNT	147.97	18.871	0.747
GO	8.70	2.197	0.001
rGO	132.50	20.571	0.504
rGO-CNT_{10}	153.48	17.446	0.324
rGO-CNT_{20}	163.42	23.100	0.481
rGO-CNT_{30}	204.12	21.327	0.575
rGO-CNT_{50}	180.95	20.570	0.536

Note: S_{BET} is the BET surface area; D_{DFT} is the DFT desorption average pore diameter; V is the total pore volume.
